# Why health recommender systems struggle to reach clinical practice: A lifecycle-oriented systematic review

**DOI:** 10.1016/j.isci.2026.116220

**Published:** 2026-06-09

**Authors:** Oumaima EL MIAYAR, Abdelaziz Berrado

**Affiliations:** 1 Research Team AMIPS, Ecole Mohammadia d’Ingénieurs, Mohammed V University in Rabat, Avenue Ibn Sina, BP 765, Agdal, Rabat, Morocco

**Keywords:** health sciences, medicine, medical specialty, health informatics

## Abstract

Health recommender systems (HRSs) are increasingly being proposed to support personalized and data-driven healthcare decisions, yet their translation into real-world practice remains limited. Existing reviews typically analyze HRSs through isolated dimensions such as algorithms, application domains, or evaluation methods, offering limited insight into why technically advanced systems rarely progress beyond prototypes. Following PRISMA 2020, we reviewed 136 peer-reviewed journal articles published between 2014 and 2025 from Scopus and PubMed. We adopted a lifecycle-oriented analytical framework, examining HRSs across six interrelated dimensions: clinical intent, data governance, recommendation logic, user interaction, evaluation strategy, and clinical integration and ethics. Our findings show that limited clinical adoption is not primarily driven by algorithmic immaturity but by persistent misalignment between intended use, evaluation design, and integration pathways. By reframing translational stagnation as a lifecycle coherence problem rather than a technical one, this review provides a unifying perspective for designing clinically credible and deployment-aware HRSs.

## Highlights

• Reviews 136 HRS studies through a lifecycle-oriented framework
• Identifies gaps between technical maturity and clinical deployment
• Shows that evaluation-deployment misalignment limits real-world translation
• Reframes HRS readiness as lifecycle coherence, not model performance

## Graphical abstract

**Figure:**
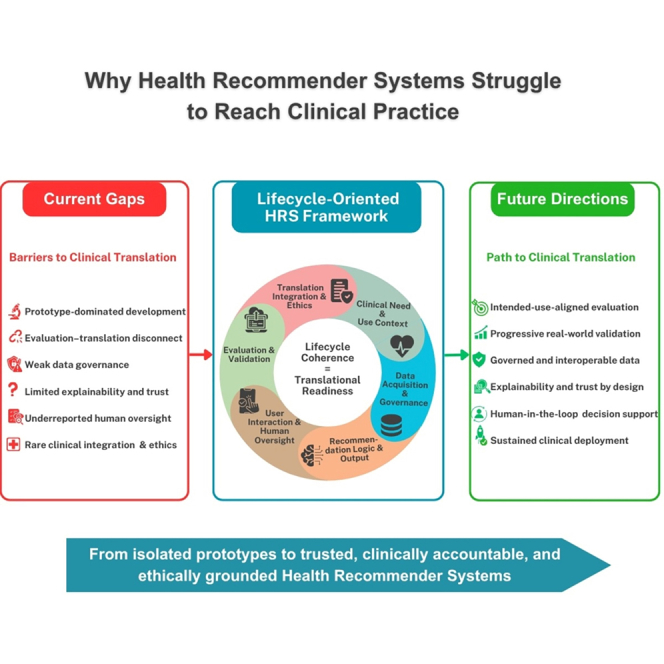

## Introduction

What should a patient recovering from a chronic illness do when new symptoms appear after hospital discharge? Should they seek immediate medical attention, adjust treatment, or wait and monitor the situation? For many individuals, navigating such decisions outside clinical settings remains fragmented and uncertain and shaped by information overload, limited follow-up, and the absence of personalized guidance.^1^ This challenge has become increasingly critical as healthcare systems shift from episodic acute care toward continuous disease management, prevention, and long-term patient self-monitoring.

In this context, personalized medicine has evolved from a conceptual ambition into a structural transformation of healthcare delivery—one that seeks to make care more proactive, precise, and responsive to individual patient profiles.^2^ Achieving this vision requires intelligent digital systems capable of integrating heterogeneous clinical and behavioral data and translating them into actionable, context-aware recommendations that support decision-making both inside and outside the clinic.

Health recommender systems (HRSs) have emerged as one such class of technologies.^3^ Building on principles from recommender systems research, HRSs aim to provide personalized guidance by recommending treatments, diagnostic pathways, preventive actions, lifestyle modifications, or follow-up strategies tailored to patients, clinicians, and care contexts.^3^ Their underlying logic spans diverse paradigms—including knowledge-based reasoning, collaborative filtering, machine learning, deep learning, graph-based models, reinforcement learning, and hybrid architectures—and their applications cover a wide range of domains, from chronic disease management and medication optimization to oncology, nutrition, mental health, rehabilitation, and early diagnosis.

Over the past decade, HRS research has experienced substantial methodological growth, driven by advances in health data availability, representation learning, and computational reasoning frameworks. These developments have enabled increasingly sophisticated systems capable of capturing complex relationships among patients, clinical conditions, interventions, and outcomes. As a result, the literature reflects considerable technical diversity and algorithmic maturity.

Yet, a striking paradox persists: while HRSs have become technically more sophisticated, very few have successfully reached routine clinical practice. Most published systems remain confined to retrospective evaluations, simulated environments, or prototype-stage demonstrations, with limited progression toward prospective validation, workflow integration, or sustained deployment in real healthcare settings. This persistent imbalance between technical advancement and translational maturity suggests that the central challenge of HRSs is not simply algorithmic performance but the absence of alignment between system design, evaluation strategy, clinical integration, and governance readiness.

Several systematic and scoping reviews have attempted to synthesize the growing HRS literature by focusing on specific dimensions such as recommendation techniques, application domains, evaluation strategies, or ethical challenges.^4^^,^^5^^,^^6^^,^^7^ As demonstrated in Table 1, these studies provide valuable insights but have largely examined HRSs through isolated analytical dimensions. Early reviews such as the systematic review by Croon et al.^4^ primarily emphasize application domains, user types, and design guidelines, offering broad descriptive taxonomies without explicitly addressing deployment maturity or translational barriers. Similarly, the review by Etemadi et al.^5^ provides a comprehensive overview of algorithms, challenges, and technical opportunities, yet evaluation, deployment, and ethics remain treated as parallel themes rather than interconnected stages of a coherent system lifecycle.

**Table 1:** Positioning of existing reviews and methodological contribution of this SLR

Review	Year	Review Type	Scope	Main axes	Key limitation	What our SLR adds
Etemadi et al.^5^	2023	systematic	general HRSs	techniques, challenges, open issues	lacks integration between evaluation, deployment, and ethics	end-to-end lifecycle alignment from design to translation
Croon et al.^4^	2021	systematic	HRSs & health applications	user types, application domains, guidelines	no analysis of deployment maturity or translational gaps	intent-aware interpretation of deployment outcomes
Sun et al.^6^	2023	scoping/evidence mapping	HRS development & evaluation	development stages, evaluation practices	evaluation independent of integration or ethics	evaluation positioned as a translational gatekeeper
Ananthakrishnan et al.^7^	2025	scoping	evaluation-focused HRSs	metrics, validation strategies	no linkage to intended use or real-world integration	cross-pillar coupling of evaluation and deployment
Figueroa et al.^8^	2025	conceptual/viewpoint	equity-oriented HRSs	fairness, socioecological factors	no systematic or lifecycle-based synthesis	ethics treated as an operational enabler of translation
Eguia et al.^9^	2024	systematic	NLP-based CDSSs	NLP techniques, CDS applications	not HRS-specific; no lifecycle or recommender focus	unified HRS-specific lifecycle perspective

Note: This table positions prior review literature relative to the analytical and lifecycle-oriented scope of the present SLR.

More recent reviews adopt narrower but deeper perspectives,^6^ with a focus on development and evaluation practices, highlighting important validation gaps and methodological trends, while the review by Ananthakrishnan et al.^7^ concentrated specifically on evaluation strategies and performance metrics. However, these analyses largely assess validation in isolation, without systematically linking evaluation depth to intended use, clinical integration pathways, or governance readiness. Complementary perspectives have also emerged from adjacent domains,^8^ emphasizing health equity and socioecological determinants of recommendation fairness, while Eguia’s^9^ review focused on NLP-based clinical decision-support systems (CDSSs). Although conceptually valuable, these studies do not provide a lifecycle-oriented synthesis centered on HRSs and their progression toward real-world implementation.

This fragmentation reveals a critical gap in the literature. Existing reviews explain how HRSs are built but provide limited understanding of why most of them fail to progress beyond experimental stages. In healthcare, predictive accuracy alone does not ensure adoption. Translation depends equally on explainability, human oversight, workflow compatibility, institutional readiness, ethical accountability, and the coherence between intended use and evaluation design. Without examining these dimensions together, technical maturity can be easily mistaken for deployment readiness.

Addressing this limitation requires moving beyond component-level analysis toward a lifecycle-oriented perspective, in which HRSs are understood as socio-technical systems whose success depends on coherence across multiple interdependent stages of design, validation, interaction, and implementation. Rather than asking whether a recommendation model performs well, the question of whether the entire system is designed to survive the realities of clinical practice becomes more consequential.

In this study, we present a systematic literature review of 136 peer-reviewed journal articles published between 2014 and 2025, adopting a lifecycle-oriented analytical framework to examine how HRSs are conceptualized, evaluated, and positioned for translation into clinical practice. The framework is organized into six complementary pillars: (1) clinical need and intended use, (2) data acquisition and governance, (3) recommendation logic and output, (4) user experience and interaction, (5) evaluation and validation strategies, and (6) translation, integration, and ethics in practice. Importantly, this framework is not proposed as a prescriptive development pipeline but as an analytical lens for identifying coherence, misalignment, and translational bottlenecks across reported system characteristics.

By systematically mapping HRSs across these six pillars and conducting cross-dimensional analyses, this review makes three principal contributions. First, it provides a comprehensive and up-to-date characterization of HRSs across technical, organizational, and translational dimensions. Second, it identifies recurrent structural misalignments—particularly between intended system use and evaluation practices—that explain why most systems remain trapped at the research prototype stage despite methodological sophistication. Third, it reframes translational readiness as a property of lifecycle coherence rather than technological maturity alone, offering a more realistic foundation for designing clinically credible and deployment-aware HRSs.

By shifting the focus from algorithmic excellence to lifecycle alignment, this review provides not only a synthesis of the existing literature but also a diagnostic lens for understanding why many HRSs struggle to reach clinical practice—and what must change for that promise to become operational reality.

## Results

### Study selection

**Figure 1:**
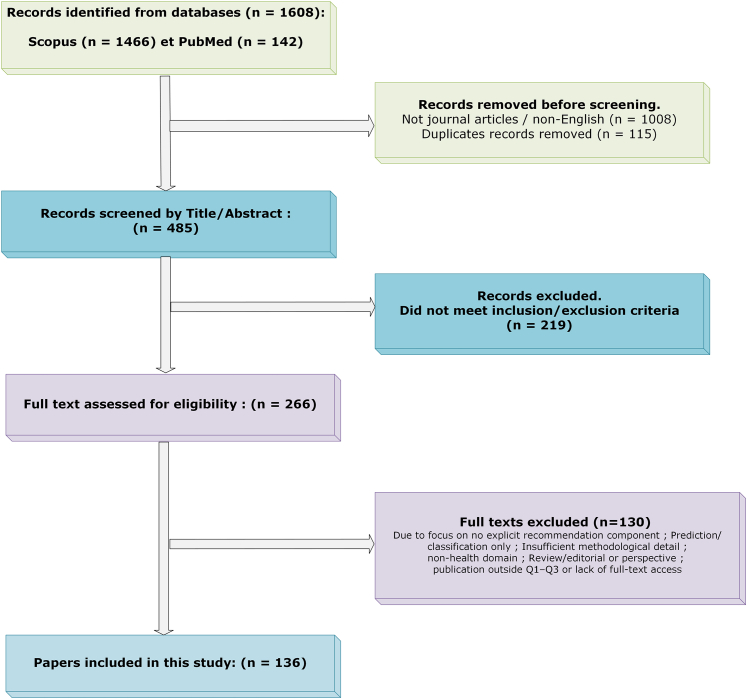
PRISMA flow diagram of the study selection process PRISMA 2020 flow diagram illustrating study identification, screening, eligibility assessment, and final inclusion. A total of 1,608 records were identified, and 136 studies were retained after screening and eligibility assessment.

The study selection process followed the PRISMA 2020 guidelines^10^ and is summarized in Figure 1. The initial search across Scopus and PubMed identified 1,608 records (1,466 from Scopus and 142 from PubMed). After applying language (English), publication type (peer-reviewed journal articles), and time restrictions (2014–2025), 600 records were retained.

Following deduplication, 485 unique articles remained for screening. Title and abstract screening excluded 219 records that did not meet the eligibility criteria, primarily due to the absence of an explicit recommendation component, non-health contexts, or a sole focus on prediction or classification tasks.

The remaining 266 articles underwent full-text assessment, leading to the exclusion of 130 studies due to insufficient methodological detail, lack of a recommender system as defined in this review, or scope misalignment. Ultimately, 136 studies met all inclusion criteria and were retained for data extraction and analysis.

### Corpus overview

This subsection provides a structured overview of the 136 included studies, focusing on the temporal evolution and publication venues. These descriptive patterns contextualize the positioning of HRSs within the broader digital health landscape.

#### Publication trends

**Figure 2:**
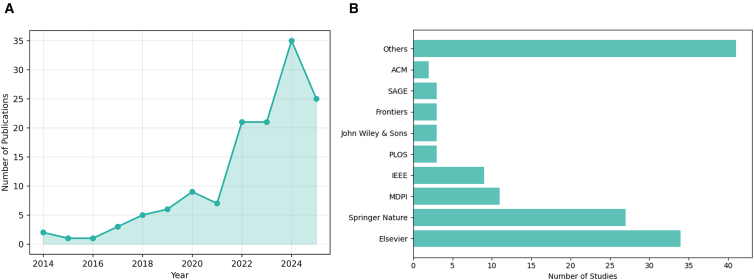
Temporal evolution and publication landscape of the included HRS studies (A) Annual publication trends of the 136 included HRS studies from 2014 to 2025, highlighting the gradual expansion of the field over time, with a marked acceleration in publication activity after 2021. (B) Distribution of the included HRS studies across major academic publishers, reflecting both the growing visibility of HRS research in established outlets and the continued dispersion of the field across heterogeneous publication venues.

The temporal distribution of publications reveals a progressive expansion of HRS research between 2014 and 2025 (Figure 2A). Early contributions (2014–2017) were limited, with one to three studies per year, reflecting an exploratory phase. From 2018 onward, publication activity increased steadily, indicating a growing methodological interest and the diversification of application domains. A marked acceleration could be observed after 2021, culminating in a peak in 2024 and sustained activity in 2025. This trajectory reflects the consolidation of HRSs as an emerging domain within digital health and personalized care.

However, this expansion in research activity is not accompanied by a corresponding shift toward higher levels of deployment. Across time, research prototypes remain predominant, with only limited progression toward pilot studies or real-world implementation. This pattern suggests that, despite increasing algorithmic sophistication and application diversity, translational maturity has not evolved at the same pace as publication growth.

#### Venue analysis

The distribution of publication venues further reflects the evolving structure of the field (Figure 2B). A substantial proportion of studies is concentrated within major scientific publishers, with Elsevier (34 papers) and Springer Nature (27) being the leading ones, followed by MDPI (11) and IEEE (9). Additional contributions are distributed across Wiley, Frontiers Media SA, SAGE Publications, ACM, and PLOS, while approximately one-third of the studies appear in a diverse range of secondary venues.

This distribution indicates increasing recognition of HRS research within the established outlets spanning artificial intelligence, biomedical informatics, and digital health. At the same time, the dispersion across multiple venues underscores the interdisciplinary nature of the field and the absence of a dominant publication hub. Overall, these patterns suggest a field in transition, moving toward greater consolidation while still being characterized by methodological diversity and evolving standards.

### General characteristics

The 136 studies included in this review exhibit substantial heterogeneity across all analyzed dimensions, reflecting the diversity of design choices and application contexts within the HRS literature. Table 2 summarizes the main study characteristics, reporting absolute counts, proportions, and two-sided 95% Wilson confidence intervals.

**Table 2:** Characteristics of the included studies (*n* = 136)

Pillar	Attribute	Category	Count (*n*)	%	95% CI
Pillar 1—Clinical need and use context	clinical domain	multi-disease	47	34.6	[27.1, 42.9]
		chronic diseases	31	22.8	[16.5, 30.5]
		general health & wellness	26	19.1	[13.4, 26.5]
		cancer	14	10.3	[6.2, 16.5]
		acute/hospital care	10	7.4	[4.0, 13.0]
		mental & neurological	4	2.9	[1.1, 7.3]
		infectious diseases	3	2.2	[0.8, 6.3]
		oral/dental health	1	0.7	[0.1, 4.0]
	clinical task (multi-label)	therapeutic recommendation	62	45.6	[37.5, 54.0]
		information, navigation, and support	40	29.4	[22.4, 37.6]
		diagnosis & risk prediction	38	27.9	[21.1, 36.0]
		prevention & lifestyle	38	27.9	[21.1, 36.0]
		monitoring & follow-up	19	13.9	[9.1, 20.8]
		research & translational discovery	2	1.5	[0.4, 5.2]
	target users	clinician-facing	52	38.2	[30.5, 46.6]
		patient-facing	35	25.7	[19.1, 33.7]
		dual-user	35	25.7	[19.1, 33.7]
		non-clinical/consumer	14	10.3	[6.2, 16.5]
	intended use	research-only	36	26.5	[19.8, 34.6]
		care-facing CDSSs	50	36.8	[29.2, 45.1]
		patient-facing health support	50	36.8	[29.2, 45.1]
Pillar 2—Data acquisition and governance	data source type	public/open data	39	28.7	[21.8, 36.8]
		retrospective clinical data	27	19.9	[14.1, 27.2]
		behavioral/patient-generated	13	9.6	[5.7, 15.6]
		sensor/IoT data	3	2.2	[0.8, 6.3]
		synthetic/simulated	6	4.4	[2.0, 9.3]
		mixed sources	46	33.8	[26.3, 42.1]
	data sensitivity	sensitive	88	64.7	[56.3, 72.2]
		non-sensitive	14	10.3	[6.2, 16.5]
		not reported	34	25.0	[18.5, 32.9]
	governance reporting	not reported	69	50.7	[42.4, 59.0]
		ethics/consent mentioned	34	25.0	[18.5, 32.9]
		regulatory compliance	7	5.1	[2.5, 10.2]
		technical governance mechanisms	26	19.1	[13.4, 26.5]
	reproducibility signal	not reported	70	51.5	[43.2, 59.7]
		data or code available	54	39.7	[31.8, 48.2]
		data and code available	9	6.6	[3.5, 12.1]
		not applicable	3	2.2	[0.8, 6.3]
Pillar 3—Recommendation logic & output	recommendation paradigm	ML/DL-based	44	32.6	[25.1, 40.6]
		hybrid	42	30.9	[23.7, 39.1]
		knowledge-based	20	14.7	[9.7, 21.6]
		graph-based	17	12.5	[8.0, 19.0]
		collaborative filtering	7	5.1	[2.5, 10.2]
		content-based	3	2.2	[0.8, 6.3]
		simulation-based	2	1.5	[0.4, 5.2]
		not reported	1	0.7	[0.1, 4.0]
	output type (multi-label)	ranked list	89	65.4	[57.1, 72.9]
		risk score/prediction	43	31.6	[24.4, 39.8]
		decision policy/care plan	36	26.5	[19.8, 34.6]
		narrative/textual advice	22	16.2	[11.0, 23.2]
		alert/flag	11	8.1	[4.6, 13.8]
		not reported	1	0.7	[0.1, 4.0]
	explainability level	not reported	81	59.6	[51.2, 67.5]
		post-hoc	13	9.6	[5.7, 15.6]
		intrinsic	17	12.5	[8.0, 19.0]
		evidence-linked	25	18.4	[12.8, 25.7]
Pillar 4—User experience and human oversight	delivery platform	not reported	87	64.0	[55.7, 71.5]
		web-based	20	14.7	[9.7, 21.6]
		mobile-based	15	11.0	[6.8, 17.4]
		web + mobile	9	6.6	[3.5, 12.1]
		EHR/CDSS-integrated	4	2.9	[1.1, 7.3]
		conversational/Chatbot-based	1	0.7	[0.1, 4.0]
	user interaction	interactive	63	46.3	[38.2, 54.7]
		passive	7	5.1	[2.5, 10.2]
		not reported	66	48.5	[40.3, 56.8]
	human-in-the-loop	none	24	17.6	[12.2, 24.9]
		review	15	11.0	[6.8, 17.4]
		final decision/override	31	22.8	[16.5, 30.5]
		not reported	66	48.5	[40.3, 56.8]
Pillar 5—Evaluation and validation	evaluation level	offline	99	72.8	[64.7, 79.7]
		mixed	20	14.7	[9.7, 21.6]
		expert validation	7	5.1	[2.5, 10.2]
		user study	3	2.2	[0.8, 6.3]
		prospective/real-world	7	5.1	[2.5, 10.2]
	outcome focus	technical	96	70.6	[62.4, 77.6]
		clinical	4	2.9	[1.1, 7.3]
		user-centered	7	5.1	[2.5, 10.2]
		mixed	29	21.3	[15.2, 29.0]
Pillar 6—Translation, integration, and ethics	clinical integration	no integration	111	81.6	[74.2, 87.2]
		partial integration	21	15.4	[10.3, 22.5]
		full integration	4	2.9	[1.1, 7.3]
	deployment stage	concept	1	0.7	[0.1, 4.0]
		research prototype	120	88.2	[81.7, 92.7]
		pilot	10	7.4	[4.0, 13.0]
		deployed	5	3.7	[1.6, 8.4]
	integration barriers (multi-label)	not discussed	61	44.9	[36.9, 53.2]
		validation gap	49	36.0	[28.4, 44.4]
		scalability	30	22.0	[15.9, 29.8]
		data/interoperability	29	21.0	[15.2, 29.0]
		workflow & adoption gap	23	16.9	[11.6, 24.0]
		regulatory & legal gap	4	2.9	[1.1, 7.3]
	ethical operationalization	not reported	58	42.6	[34.6, 51.0]
		mentioned only	34	25.0	[18.5, 32.9]
		partially operationalized	32	23.5	[17.1, 31.4]
		explicitly operationalized	12	8.8	[5.1, 14.8]

Summary of extracted attributes (counts, percentages, and two-sided 95% Wilson confidence intervals).

Counts are reported over the full corpus (*n* = 136). Percentages and two-sided 95% Wilson confidence intervals are computed relative to *n* = 136; attributes marked as multi-label are not mutually exclusive and may sum to more than 100%.

Studies span a wide range of clinical domains, recommendation paradigms, data sources, target users, evaluation strategies, and levels of clinical integration. Most attributes were coded as single label. However, clinical tasks, recommendation output types, and reported integration barriers were treated as multi-label dimensions, as individual systems frequently address multiple tasks, generate more than one form of output, or report several concurrent barriers. Percentages for these attributes are, therefore, non-mutually exclusive and should be interpreted descriptively.

Overall, the corpus reflects a methodologically diverse field with uneven maturity across the HRS lifecycle, where systems often combine multiple design elements while remaining at early stages of evaluation and real-world integration. Subsequent sections analyze these dimensions in detail, using the proposed pillar-based framework.

### Lifecycle-oriented findings across six analytical pillars

To provide a structured synthesis of the reviewed HRSs, this section analyzes system characteristics through six complementary analytical pillars reflecting the HRS lifecycle. Rather than examining design choices in isolation, these pillars capture how clinical intent, data foundations, recommendation logic, user interaction, evaluation strategies, and real-world integration jointly shape system maturity.

The framework is operationalized through twenty explicitly defined attributes, with standardized definitions and coding values detailed in Table S1. By examining these dimensions jointly, the analysis reveals recurrent design patterns, reporting gaps, and translational discontinuities that would remain obscured in attribute-by-attribute assessments, offering an integrated perspective on how—and how far—HRSs have progressed toward clinically accountable use.

#### Clinical need and use context

At the entry point of the HRS lifecycle, this pillar examines how systems define their clinical scope and intended use. It characterizes the targeted medical domains, supported clinical tasks, primary user groups, and operational contexts, thereby clarifying not only which healthcare needs are addressed but also how these needs are framed and operationalized within decision-making processes.

##### Clinical domains and scope of application

Across the reviewed corpus (*n* = 136), HRSs are deployed across a wide range of clinical domains, reflecting both the versatility of recommender technologies and an uneven distribution of research focus.

A dominant share of systems is designed for multi-disease settings (34.6%), indicating a strong emphasis on generalizable architectures capable of operating across heterogeneous patient populations (Table 2). While such approaches promote scalability and cross-condition applicability, they often rely on abstracted patient representations and shared knowledge structures, which may limit their capacity to capture disease-specific clinical nuances.^11^^,^^12^^,^^13^ This suggests a structural trade-off between generalizability and clinical specificity in the current HRS design.

Chronic disease contexts account for 22.8% of the corpus, reflecting a natural alignment between recommender systems and longitudinal care scenarios.^14^^,^^15^ In these settings, repeated decision-making, continuous monitoring, and adaptive treatment strategies create favorable conditions for recommendation-based support.^16^^,^^17^ Accordingly, HRSs targeting chronic conditions frequently emphasize ongoing guidance and dynamic adaptation, positioning recommendations as integral components of long-term care management rather than isolated decision aids.^18^^,^^19^

General health and wellness applications represent 19.1% of the studies, illustrating the expansion of HRSs beyond formal clinical environments. These systems are typically embedded in preventive and lifestyle-oriented contexts, where personalization is driven by user preferences and self-reported data rather than strict clinical protocols.^20^^,^^21^^,^^22^^,^^23^ This shift reflects a broader trend toward proactive and user-centered healthcare.

In contrast, cancer-focused systems account for 10.3% of the corpus and are predominantly situated in high-complexity clinical settings.^24^ These systems often address critical tasks such as treatment selection or therapy planning and rely heavily on structured clinical knowledge, expert-driven rules, or knowledge graphs.^25^^,^^26^ Their relatively limited representation suggests that, despite high clinical relevance, the development of HRSs in oncology remains constrained by data sensitivity, methodological complexity, and regulatory requirements.

Other clinical contexts appear less frequently in the literature. Acute and hospital care settings account for 7.4% of the corpus, while mental and neurological health (2.9%), infectious diseases (2.2%),^27^ and oral and dental health (0.7%)^28^ remain underrepresented in the literature. Although these domains are clinically significant, their limited presence may be attributed to challenges related to data availability, evaluation complexity, and regulatory constraints. Taken together, this distribution underscores uneven coverage across clinical domains and points to underexplored opportunities for HRS development in specialized and acute-care settings.

##### Clinical tasks and functional scope

Clinical tasks define the functional role of HRSs within care processes. Unlike conventional decision-support systems that typically target a single objective, HRSs frequently span multiple interdependent tasks across the clinical decision lifecycle.^29^^,^^30^ To capture this structural complexity, tasks were modeled as a multi-label attribute.

**Figure 3:**
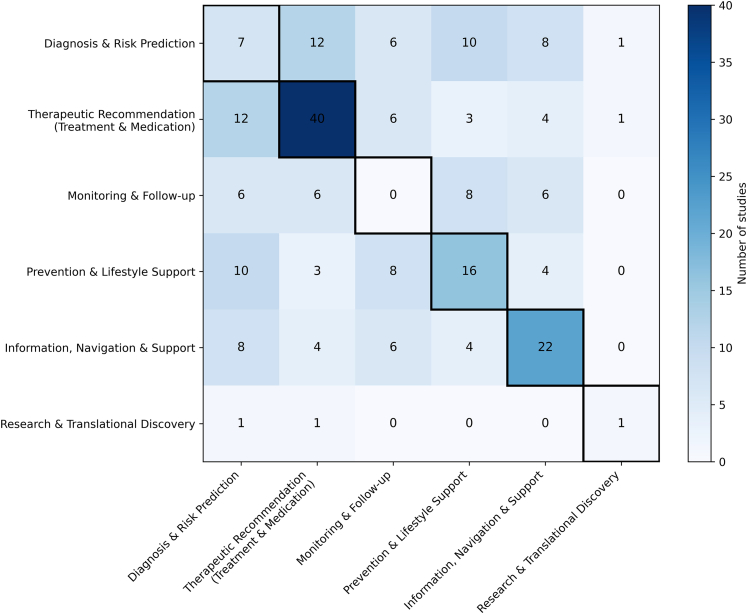
Clinical task coupling matrix in HRSs (*n* = 136) The heatmap represents the co-occurrence frequency between clinical task categories across the included studies. Rows and columns represent clinical task categories (multi-label). Diagonal cells indicate studies addressing a single clinical task, whereas off-diagonal cells represent multi-task coupling. Cell values correspond to the number of studies.

The resulting task organization, summarized through a co-occurrence matrix (Figure 3), reveals a clear combination of dominant standalone functions and recurrent multi-task configurations. Therapeutic recommendation emerges as the primary standalone task (*n* = 40),^31^ reflecting the centrality of treatment-related decisions in clinical practice. In contrast, diagnosis and risk prediction (*n* = 7)^32^ and prevention and lifestyle support (*n* = 16)^33^ rarely appear in isolation, indicating that they are typically embedded within broader decision-support pipelines rather than being treated as independent endpoints.

More importantly, consistent patterns of task coupling reveal a clinically coherent but asymmetrically structured workflow. Diagnosis and risk prediction systematically operate as an upstream component, informing downstream tasks such as therapeutic recommendation (*n* = 12),^34^ prevention (*n* = 10),^35^ and informational support (*n* = 8).^36^ This configuration reflects a pipeline-oriented logic in which inference, action, and support are tightly interconnected.

Monitoring and follow-up appear predominantly as a complementary function, most often associated with prevention (*n* = 8)^37^ and informational support (*n* = 6).^38^ Its limited presence as a standalone task highlights its inherently longitudinal role, but it also reveals a structural limitation in current HRS design, where continuous care processes are rarely treated as primary recommendation objectives. Similarly, prevention occupies an intermediate position, bridging predictive and therapeutic functions and reflecting a shift toward anticipatory care paradigms, although its integration remains uneven across systems.

Information and navigation support functions exhibit a transversal pattern, co-occurring with most task categories. Rather than serving as primary objectives, they act as enabling layers that enhance interpretability, user engagement, and decision comprehension. This widespread integration suggests that explainability and communication are increasingly being embedded within HRSs, although often implicitly rather than through explicit design frameworks.

In contrast, research-oriented or discovery-focused tasks remain marginal and weakly connected to other functions,^34^ indicating that HRSs are predominantly positioned as applied decision-support tools rather than exploratory systems. This limited integration of discovery-oriented functions may constrain the potential of HRSs to contribute to knowledge generation and adaptive learning in clinical settings.

Overall, HRSs are structured around a limited set of dominant functions, with therapeutic and diagnostic tasks forming the core of most systems. While multi-task configurations reflect realistic clinical workflows, the uneven integration of monitoring, prevention, and research functions reveals structural gaps that may limit system comprehensiveness, longitudinal adaptability, and ultimately real-world applicability.

##### Target users and intended use

Beyond clinical domain and task orientation, target users and intended use provide essential insight into how HRSs are positioned within healthcare ecosystems. Together, these dimensions clarify for whom recommendations are designed and under what assumptions regarding responsibility, decision authority, and operational context. In this review, both attributes were treated as single-label variables, capturing the primary audience and intended functional role explicitly stated by the authors.

Clinician-facing systems constitute the largest category (38.2%) (Table 2), highlighting a strong emphasis on supporting professional decision-making. These systems are typically embedded in diagnostic or therapeutic contexts and position recommendations as advisory inputs within expert-driven workflows.^31^^,^^32^ Their prominence reflects a strong orientation toward formal care processes, although such positioning does not necessarily imply real-world integration.

Patient-facing systems account for 25.7% of the corpus and emphasize accessibility, personalization, and user engagement. These systems are commonly applied in chronic disease management and wellness contexts, where recommendations support self-management and behavioral change.^39^^,^^40^^,^^41^^,^^42^ Their growth reflects a broader shift toward patient empowerment, although it also introduces challenges related to usability, trust, and adherence.

Dual-user systems represent an equivalent share (25.7%), pointing to an emerging design paradigm in which HRSs are intended to support both clinicians and patients within shared decision-making processes.^43^^,^^44^^,^^45^^,^^46^ By bridging professional expertise and patient participation, these systems align conceptually with collaborative care models. At the same time, they introduce additional complexity in terms of interface design, responsibility allocation, and consistency of recommendations across user groups.

In contrast, non-clinical or consumer-facing systems remain limited (10.3%), suggesting that HRSs are more often framed as health-related decision-support tools than as purely consumer-oriented recommendation applications.^47^^,^^48^ These systems are generally oriented toward general health information, wellness support, or service navigation outside formal care settings.

The intended-use dimension further refines this landscape by clarifying the operational role envisioned for HRSs (Table 2). Research-oriented systems account for 26.5% of the corpus, representing a substantial subset of studies developed primarily as methodological proofs of concept. These systems emphasize algorithmic innovation and performance evaluation without explicit claims of deployment, user-facing operation, or integration into care processes.^49^^,^^50^ Their positioning is consistent with exploratory research objectives and differs fundamentally from systems designed for real-world clinical or patient-facing use.

Care-facing CDSSs form the largest intended-use category (36.8%), indicating that many HRSs are conceptually aligned with clinical practice.^51^^,^^52^ However, this alignment often remains aspirational, as a considerable proportion of these systems lack evidence of deployment, validation in clinical environments, or integration into operational workflows. This reveals a recurring tension between the stated purpose and actual system maturity.

Patient- or consumer-facing health support systems also account for 36.8% of the reviewed studies.^53^^,^^54^ Their strong presence reflects a parallel trajectory centered on personalization, accessibility, and user engagement. However, these systems operate under different constraints compared to clinician-facing applications, particularly in terms of validation rigor, risk tolerance, and responsibility for decision-making.

Overall, most HRSs clearly define their target users and intended role, reflecting a conceptually structured design landscape. However, this clarity is not matched by equivalent translational maturity, as many systems remain confined to research settings or early-stage prototypes. This suggests that defining user and use context, while necessary, is not sufficient for real-world impact. Translational progress depends on alignment with appropriate evaluation strategies, interaction design, and pathways toward clinical integration, as examined in subsequent sections.

#### Data acquisition and governance

This pillar examines the data foundations underpinning HRSs and the extent to which governance, transparency, and reproducibility are explicitly reported. Rather than cataloging datasets, the analysis focuses on how data origin, sensitivity, and reporting practices jointly shape trust, accountability, and translational readiness. In particular, this perspective highlights how technical design choices related to data acquisition are intrinsically linked to broader issues of transparency and deployment feasibility.

##### Data foundations and sensitivity patterns

HRSs rely on diverse data sources, including public datasets (28.7%),^55^^,^^56^ retrospective clinical data (19.9%),^57^^,^^58^ patient-generated behavioral data (9.6%),^57^^,^^59^ sensor or IoT data (2.2%),^60^ synthetic data (4.4%),^61^^,^^62^ and mixed data sources (33.8%).^63^^,^^64^^,^^65^ The prominence of mixed data reflects a clear shift toward multi-source integration, where clinical, behavioral, and contextual information are combined to support more personalized and context-aware recommendation processes.

Across the corpus, most systems (64.7%) rely on sensitive data, while only a limited fraction (10.3%) uses non-sensitive data. Notably, 25.0% of studies do not report data sensitivity, restricting transparency and limiting the assessment of data-related risks. Although non-reporting does not imply the absence of protection mechanisms, it introduces uncertainty regarding how data sensitivity is handled and communicated.

**Figure 4:**
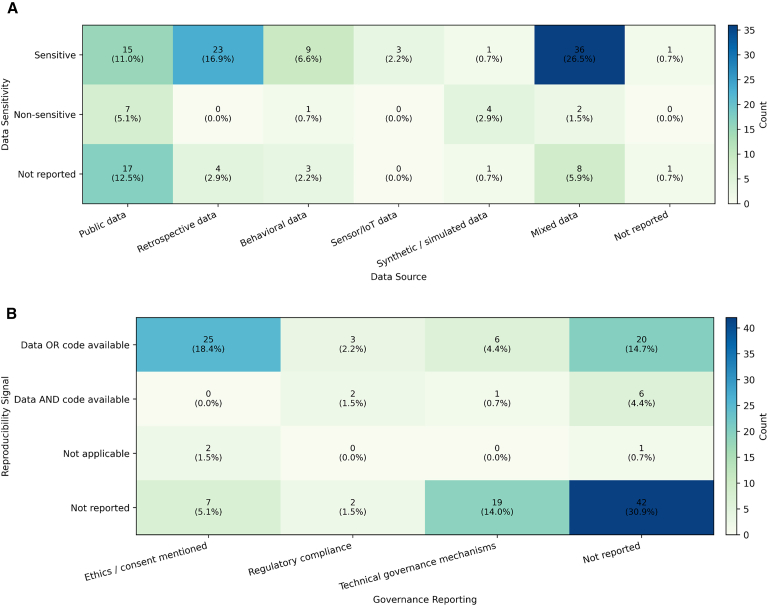
Data foundations, sensitivity, and transparency patterns in HRSs (*n* = 136) (A) Heatmap showing the relationship between data source categories and reported data sensitivity levels. The distribution highlights the predominance of sensitive and multi-source data. (B) Heatmap showing the relationship between governance reporting categories and reproducibility signals. The distribution reveals substantial gaps in transparency and reporting practices. Both (A) and (B) indicate a structural imbalance: increasing reliance on sensitive data is not matched by consistent governance or reproducibility practices.

As illustrated in Figure 4A, sensitivity patterns vary systematically across data sources. Mixed and retrospective clinical data are predominantly associated with sensitive information, confirming the central role of patient-level data aggregation in HRS design. Sensor-based data, although less frequent, are almost exclusively sensitive due to their continuous and highly personal nature, often capturing real-time physiological or behavioral signals. Public datasets span all sensitivity categories, highlighting that accessibility does not imply reduced sensitivity^66^^,^^67^ and that publicly available data may still require strict governance.

Taken together, these findings reveal a structural dependency on sensitive, multi-source data that is not consistently accompanied by explicit sensitivity reporting. This gap not only limits transparency but also constrains the evaluation of data governance practices and the assessment of system readiness for real-world deployment.

##### Governance, reproducibility, and transparency

Governance reporting and reproducibility signals remain highly uneven across the corpus. Over half of the studies have not reported governance mechanisms (50.7%) or reproducibility signals (51.5%), indicating substantial limitations in transparency. These indicators reflect reporting practices rather than the actual presence of governance or reproducibility mechanisms, yet they remain critical for assessing scientific rigor and trustworthiness.

As shown in Figure 4B, the absence of governance reporting frequently coincides with the absence of reproducibility signals. The largest share of studies (30.9%) falls into the combined “not reported governance/not reported reproducibility” category, pointing to a systemic transparency gap, rather than isolated reporting omissions.^68^^,^^69^

Ethics or consent statements are reported in a minority of studies and are most often associated with partial reproducibility, without consistently reaching higher transparency levels.^70^ Similarly, technical governance mechanisms—such as privacy-preserving architectures or access control strategies—have been infrequently reported and typically coexist with incomplete reproducibility disclosure. Regulatory compliance (e.g.,General Data Protection Regulation (GDPR) and Health Insurance Portability and Accountability Act (HIPAA)) remains rare and weakly connected to reproducibility practices, suggesting that transparency and regulatory readiness are not systematically aligned in current HRS research.

Although some studies have provided data or code, a very limited subset has reported both, representing the highest level of reproducibility.^18^^,^^29^^,^^71^ While the absence of reporting does not imply the absence of underlying practices, it significantly limits the assessment of methodological rigor, transparency, and accountability from a scientific perspective.

Taken together, these findings reveal a critical structural imbalance in the HRS landscape: the increasing reliance on sensitive, patient-level data is not matched by consistent governance reporting or reproducibility practices. This misalignment undermines transparency, weakens accountability, and ultimately limits the readiness of HRSs for safe and effective real-world clinical deployment.

#### Recommendation logic and output

This pillar examines how HRSs operationalize internal decision-making processes, focusing on the underlying recommendation paradigms, the nature of generated decision artifacts, and the extent to which algorithmic reasoning is made explicit. Together, these elements determine how recommendations are inferred, communicated, and ultimately acted upon in healthcare settings.

##### Recommendation paradigms

Across the reviewed corpus (*n* = 136), HRSs exhibit a diverse range of recommendation paradigms, reflecting heterogeneous approaches to modeling and justifying decisions. As shown in Table 2, the literature is dominated by data-driven paradigms, with machine learning and deep learning (ML/DL) approaches accounting for 32.6% of studies,^70^^,^^72^^,^^73^ closely followed by hybrid systems (30.9%) that explicitly combine multiple paradigms.^74^^,^^75^^,^^76^^,^^77^ This predominance reflects current research practices and the growing availability of high-dimensional health data, rather than a definitive methodological preference.

ML/DL-based systems primarily rely on pattern-driven inference, where predictive performance is achieved through the extraction of latent relationships from clinical, behavioral, or contextual data.^52^^,^^78^ In such settings, recommendation logic is embedded within model representations and optimization processes, rather than being expressed through explicit rules. The strong presence of hybrid systems further suggests that purely data-driven approaches are frequently complemented with additional reasoning layers—such as domain knowledge, similarity modeling, or graph structures—to address limitations related to interpretability, data sparsity, and contextual awareness.^79^^,^^80^

Knowledge-based systems (14.7%) remain an important component of the HRS landscape, operationalizing recommendation logic through clinical rules, ontologies, or guideline-driven reasoning.^81^^,^^82^^,^^83^^,^^84^ Their continued presence highlights the relevance of explicit and controllable reasoning frameworks, particularly in contexts where transparency is critical. Similarly, graph-based approaches (12.5%) model relationships between patients, conditions, and treatments, framing recommendation as an inference task over structured clinical knowledge spaces.^85^^,^^86^

In contrast, collaborative filtering (5.1%) and content-based approaches (2.2%) are less prevalent, likely due to challenges related to patient heterogeneity, sparse preference signals, and the contextual complexity of healthcare data.^87^^,^^88^^,^^89^ Simulation-based paradigms remain marginal (1.5%), indicating that explicit modeling of clinical trajectories has been rarely used as a primary recommendation mechanism.^90^^,^^91^

Overall, HRSs are rarely grounded in a single paradigm. Instead, the field is characterized by a convergence of data-driven, knowledge-based, and structure-aware approaches, reflecting ongoing efforts to balance predictive performance, interpretability, and domain constraints. These paradigm choices directly shape the form and actionability of the outputs generated by HRSs.

##### Output semantics and decision artifacts

HRSs translate internal reasoning into a range of decision artifacts that define how recommendations are communicated and interpreted. Output types were coded independently because a given HRS may generate multiple output forms, reflecting the complexity of healthcare decision-making.

Ranked lists are the most prevalent output form (65.4%), indicating that many systems frame recommendation as a prioritization task, where candidate options—such as treatments or services—are ordered by relevance.^39^^,^^79^^,^^92^^,^^93^^,^^94^ These outputs support comparative judgment rather than prescriptive decisions, positioning users as the final decision-makers.

Predictive outputs (31.6%), including risk scores and probability estimates, highlight the central role of outcome prediction in HRS design.^95^ These outputs translate model inference into quantitative indicators that support risk stratification and decision comparison.^96^^,^^97^ In this context, recommendation is framed as an evaluative process grounded in prediction rather than direct action selection.

More structured outputs, such as decision policies or care plans (26.5%), encode a higher level of semantic commitment by aggregating multiple signals into coherent recommendations.^52^^,^^98^^,^^99^^,^^100^ These approaches move beyond isolated suggestions toward integrated representations of clinical action, often within reinforcement learning or causal inference frameworks.

Textual or narrative advice (16.2%) is typically used to contextualize or explain recommendations, particularly in behavioral or preventive care settings.^40^^,^^101^^,^^102^ Alert-based outputs remain less common (8.1%), generally serving as complementary signals rather than primary recommendation mechanisms.^103^

Overall, HRSs predominantly rely on prioritization and prediction, occasionally enriched with structured or explanatory outputs. This configuration reflects a tendency to support decision-making through interpretable and graded signals rather than rigid prescriptions.

##### Explainability as a property of the decision logic

Explainability plays a central role in shaping how recommendation logic is interpreted and trusted. In this review, explainability is assessed based solely on what is explicitly reported in the studies, reflecting design articulation rather than inherent model properties.

A substantial proportion of studies (59.6%) have not reported any explainability mechanism, limiting the transparency of the underlying decision process. Post-hoc explainability methods are reported in 9.6% of the corpus, while intrinsic explainability accounts for 12.5%, and evidence-linked explanations—grounded in clinical knowledge—represent 18.4% (Table 2).

**Figure 5:**
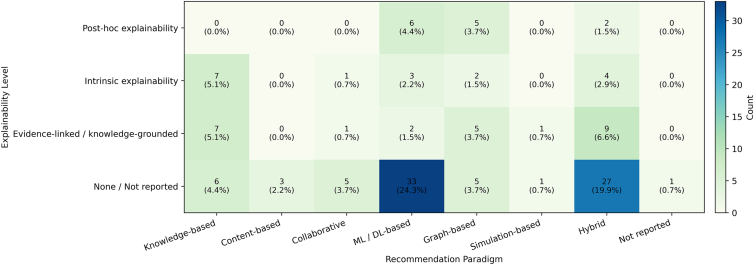
Explainability level and recommendation paradigm in HRSs (*n* = 136) The heatmap illustrates the distribution of explainability levels across recommendation paradigms. ML/DL-based and hybrid systems are largely associated with absent or unreported explainability, whereas knowledge- and graph-based approaches more frequently provide intrinsic or evidence-linked explanations. These patterns reveal a structural imbalance between the widespread use of data-driven methods and the limited transparency of their decision-making processes.

As illustrated in Figure 5, reporting patterns vary across paradigms. ML/DL-based systems are predominantly associated with absent or under-reported explainability,^104^ reflecting the implicit nature of representation learning. In contrast, knowledge-based and graph-based systems more frequently support intrinsic or evidence-linked explanations, as their reasoning structures are explicitly encoded.^105^^,^^106^ Hybrid systems exhibit heterogeneous explainability profiles, reflecting the diversity of their architectural compositions.^107^^,^^108^

Overall, explainability remains unevenly integrated across HRSs and is often treated as an optional rather than a foundational design dimension. This limitation raises important concerns for trust, accountability, and adoption, particularly in clinical contexts where recommendations may inform high-stakes decisions.^109^

#### User experience, interaction, and human oversight

While previous pillars examine clinical context, data foundations, and recommendation logic, this pillar focuses on how HRSs interface with human users and how decision authority is distributed between algorithmic components and human actors. It considers delivery platforms, interaction modalities, and human-in-the-loop (HITL) configurations as key socio-technical dimensions that shape system usability, accountability, and translational readiness.

##### Delivery platforms and interaction modalities

HRSs are not solely defined by their computational models but also by how recommendations are delivered and operationalized in practice. Delivery platforms and interaction modalities jointly determine whether recommendations remain analytical outputs or evolve into actionable decision-support tools. As such, these dimensions are inherently coupled, with the deployment context directly constraining the forms of interaction that can be supported.

Across the reviewed corpus (*n* = 136), delivery platforms remain substantially underreported, with 64.0% of studies not specifying how recommendations are deployed (Table 2). Among reported cases, web-based platforms (14.7%) and mobile applications (11.0%) dominate, followed by hybrid web-mobile solutions (6.6%). In contrast, integration within electronic health records (EHRs) or CDSSs is limited (2.9%), and conversational interfaces remain marginal (0.7%). This distribution suggests that many HRSs have been developed in experimental or prototype settings, rather than being embedded within established clinical workflows.^102^^,^^110^

A comparable pattern is observed for interaction modalities. Nearly half of the studies (48.5%) have not reported how users interact with the system,^111^^,^^112^^,^^113^ while 46.3% describe interactive designs,^114^ and only 5.1% have relied on passive interaction modes.^83^ This absence primarily reflects reporting limitations rather than a lack of interaction by design, but, nonetheless, constrains the assessment of user experience.

**Figure 6:**
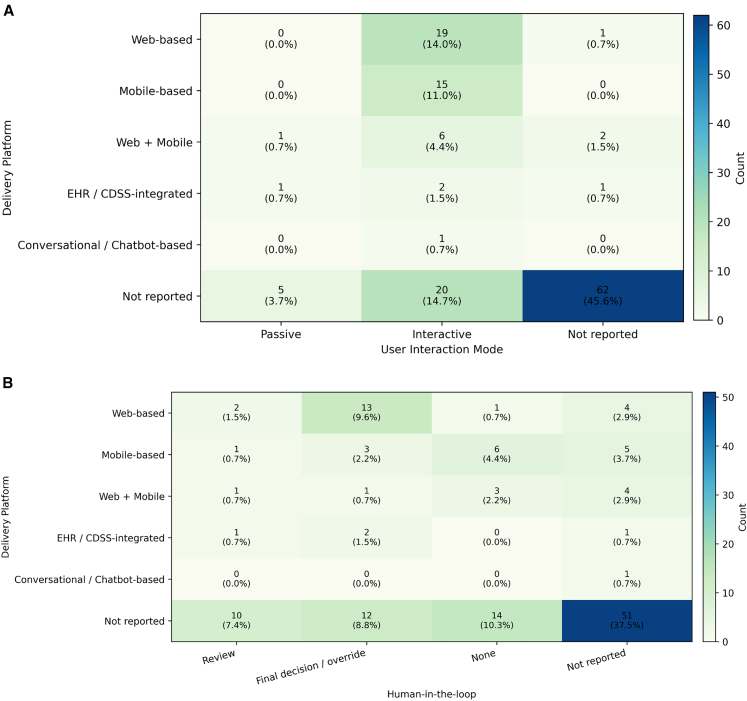
Platform-mediated interaction and decision authority in HRSs (*n* = 136) (A) Heatmap showing the relationship between delivery platforms and user interaction modes. The distribution highlights the predominance of interactive designs when platforms are explicitly reported, while a large proportion of studies remain unspecified. (B) Heatmap showing the relationship between delivery platforms and human-in-the-loop configurations. The distribution indicates that human oversight is more frequently articulated when deployment context is defined, whereas “not reported” categories remain dominant. (A) and (B) reveal a consistent reporting pattern: the absence of platform specification is strongly associated with missing information on both interaction and decision authority, pointing to persistent gaps in the documentation of user experience and governance in HRSs.

Consistent patterns emerge when jointly examining delivery platforms and interaction modalities (Figure 6A). Systems with unspecified platforms overwhelmingly coincide with unreported interaction modes, indicating that documentation gaps tend to co-occur across both dimensions. When platforms are explicitly described, interactive interaction dominates across web-based, mobile, and hybrid systems, suggesting that user engagement is typically considered when deployment context is articulated.^40^^,^^101^ Conversational systems, although rare, are almost exclusively interactive, while EHR/CDSS-integrated systems more consistently report structured interaction, reflecting the formalized nature of clinical environments.^115^

Overall, while interactive engagement appears to be a common design intention, its implementation remains insufficiently documented. This limits the ability to assess how recommendations are experienced by end-users and constrains the evaluation of system maturity beyond algorithmic performance.

##### HITL and decision authority

Beyond interface-level interaction, HITL configurations play a defining role in how decision authority and responsibility are structured within HRSs. In healthcare settings, HITL mechanisms extend beyond user interaction, determining how recommendations are validated, interpreted, or overridden within clinical or care-related processes.^116^

In this review, HITL is conceptualized as a spectrum of decision authority, rather than a binary attribute. Systems are categorized based on whether no human involvement is reported, human review or interpretation is required, or final decision authority and override capability are explicitly assigned to a human actor. These configurations reflect varying degrees of delegation between algorithmic inference and human judgment, with direct implications for accountability and governance.

As shown in Table 2, explicit reporting of HITL remains limited. Nearly half of the studies (48.5%) have not specified any form of human involvement. Among reported configurations, 11.0% describe human review or interpretation, while 22.8% explicitly assign final decision authority to a human actor. A smaller subset (17.6%) reports no human involvement based on authors’ descriptions, positioning the system as autonomous within the reported design.

The absence of reported HITL mechanisms should be interpreted as a limitation in documentation, rather than as evidence of fully autonomous deployment. In clinical contexts, such omissions obscure critical aspects of decision governance, including responsibility allocation and liability structures.^31^ As a result, it becomes difficult to determine whether systems function as advisory tools or as decision-driving components.

When examined in relation to delivery platforms (Figure 6B), clearer patterns emerged in systems with explicitly reported deployment contexts. EHR/CDSS-integrated systems more frequently assign final decision authority to human actors, consistent with established clinical accountability frameworks. In contrast, web-based and mobile systems are more commonly associated with human review configurations, aligning with their role as decision-support interfaces rather than autonomous decision-makers.

Taken together, these findings indicate that HITL mechanisms remain unevenly articulated despite their central role in shaping accountability and real-world integration. While human oversight is often implicitly assumed, its explicit operationalization is rarely detailed, limiting the assessment of governance maturity and translational readiness. This ambiguity further complicates the distinction between advisory and decision-driving systems, a critical factor for the safe deployment of HRSs in clinical environments.^25^

#### Evaluation and validation strategies

This pillar examines how HRSs are evaluated and validated, and how these choices implicitly position systems along the research-to-practice continuum. Evaluation strategies provide a critical lens on whether HRSs remain algorithmic prototypes or evolve toward clinically accountable decision-support tools.

Evaluation choices signal whether HRSs are conceived primarily as algorithmic prototypes, decision-support tools, or clinically actionable systems. Across the reviewed corpus (*n* = 136), evaluation practices remain predominantly algorithm centric, with offline algorithmic evaluation constituting the dominant validation approach (72.8%).^117^^,^^118^^,^^119^^,^^120^ Table 2 highlights the overwhelming predominance of offline evaluation, revealing a sharp discontinuity between algorithm-level validation and real-world assessment.

This reliance on offline evaluation reflects both practical feasibility constraints and a prevailing conception of HRSs as technical artifacts, rather than socio-technical systems embedded in care delivery contexts. Offline validation plays a crucial role in early-stage development, comparative benchmarking, and proof-of-concept evaluation. However, by design, it provides limited insight into how recommendations are interpreted, acted upon, or integrated into clinical workflows and does not account for human factors, contextual constraints, or safety considerations.

More advanced evaluation strategies remain comparatively scarce. Mixed evaluation designs—combining technical metrics with expert review, limited user feedback, or constrained real-world testing—have been reported in 14.7% of the studies.^33^ These hybrid approaches typically represent transitional evaluation stages, where algorithmic performance is supplemented by domain expertise or preliminary usability considerations. In contrast, expert-based validation (5.1%),^121^ user studies (2.2%),^122^ and prospective or real-world evaluations (5.1%) together constitute only a small fraction of the corpus.^115^ Importantly, these higher-fidelity evaluation strategies have almost exclusively been observed in systems explicitly positioned as care facing or patient facing, suggesting that the evaluation depth is closely aligned with the declared system intent.^116^ Research-oriented systems, by contrast, overwhelmingly rely on offline validation, reinforcing a structural separation between methodological innovation and translational ambition (Table 2).

A parallel imbalance is observed in the types of outcomes assessed. Technical outcomes dominate the literature (70.6%), with a strong emphasis on predictive accuracy, ranking metrics, loss optimization, or computational efficiency.^78^^,^^123^ Table 2 illustrates this pronounced skew toward technical performance indicators, highlighting the relative marginalization of clinical and user-centered endpoints. Explicit clinical outcomes—such as impact on decision quality, care processes, or patient safety—have been reported in only 2.9% of the studies,^29^ while user-centered outcomes, including usability or satisfaction measures, appear in 5.1% of the corpus.^124^ A subset of studies (21.3%) have adopted mixed-outcome frameworks, attempting to bridge the algorithmic performance with human or clinical relevance, but these remain the exception rather than the norm.

Taken together, the combined dominance of offline evaluation strategies and technical outcome focus reveals a structural evaluation gap within HRS research. While algorithmic performance has been extensively characterized, evidence regarding real-world effectiveness, usability, accountability, and clinical impact remains limited. This imbalance does not necessarily indicate a lack of clinical relevance by design, but rather reflects structural, regulatory, and resource-related barriers to conducting prospective evaluations in healthcare settings, including data access constraints, ethical approval requirements, and deployment complexity.

Overall, these findings suggest that evaluation strategies constitute a critical bottleneck in the translation of HRSs from experimental systems to clinically accountable decision-support tools. Alignment between evaluation level, outcome focus, and intended system use emerges as a necessary—yet inconsistently realized—condition for translational readiness. These observations set the stage for the following pillar, which examines how clinical integration, deployment practices, and ethical considerations are addressed in real-world HRS implementations.

#### Translation, integration, and ethics in practice

This pillar examines how HRSs transition from research artifacts to clinically integrated and ethically operationalized systems. The focus is on real-world positioning, including clinical integration, deployment stage, integration barriers, and the operationalization of ethical and governance mechanisms.

##### Clinical integration and deployment reality

Clinical integration and deployment are key indicators of the translational maturity of HRSs, reflecting the transition from experimental settings to operational clinical use.^43^ In this study, translational maturity is defined as the degree of alignment between system design, evaluation, and real-world implementation. Deployment stages were categorized based on real-world exposure and operational use. “Concept systems” refer to early-stage designs without empirical validation. “Research prototypes” correspond to systems evaluated in offline or simulated environments without real user interaction. “Pilot systems” denote limited real-world testing in controlled settings, while “deployed systems” are integrated into routine workflows and support real decision-making.

Across the reviewed corpus (*n* = 136), most systems remain far from operational care environments. As summarized in Table 2, 81.6% of HRSs report no clinical integration, indicating that recommendations are neither embedded in clinical workflows nor evaluated *in situ*.^125^^,^^126^^,^^127^^,^^128^^,^^129^^,^^130^ Partial integration (15.4%) typically involves expert validation, retrospective testing on clinical data, or limited workflow alignment without live system embedding.^117^ Only 2.9% of systems report full clinical integration, underscoring the rarity of HRSs incorporated into routine care processes.

A similarly skewed distribution is observed across deployment stages. The majority of systems are classified as research prototypes (88.2%),^131^^,^^132^^,^^133^ reflecting proof-of-concept implementations without real-world interaction. Pilot deployments remain limited (7.35%), while fully deployed systems account for only 3.68%, indicating that a few HRSs progress to sustained operational use.

**Figure 7:**
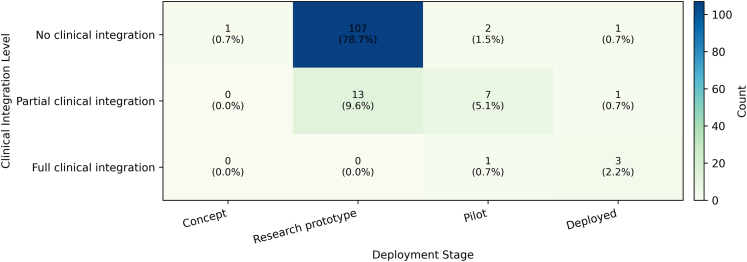
Clinical integration level and deployment stage in HRSs (*n* = 136) Heatmap showing the joint distribution of clinical integration levels and deployment stages. The results highlight a strong concentration of research prototypes without clinical integration, while pilot and deployed systems are primarily associated with higher levels of integration. Overall, the distribution reveals that deployment progression and clinical integration remain partially decoupled, reinforcing the presence of a structural translation gap in HRSs.

When examined jointly (Figure 7), clinical integration and deployment stage exhibit partially decoupled trajectories. The dominant configuration corresponds to systems with no clinical integration deployed as research prototypes (78.7%), confirming that most HRSs remain confined to controlled environments. Partial integration appears primarily in prototype and pilot settings, suggesting exploratory engagement with clinical contexts that does not systematically translate into deployment progression. Full clinical integration is observed only at advanced stages, mainly in pilot (0.7%) and deployed systems (2.2%),^26^^,^^116^ reflecting the substantial technical, organizational, and regulatory efforts required to achieve both deployment and meaningful clinical embedding.

Taken together, these findings reveal a structural translation gap. While methodological advances in HRSs are substantial, they are rarely accompanied by equivalent progress toward clinical integration or sustained deployment. Clinical integration and deployment, thus, emerge as related but not sequential dimensions of system maturity, challenging the assumption of a linear transition from prototype to real-world adoption.

##### Integration barriers and ethical operationalization

Understanding why HRSs fail to achieve sustained clinical integration requires examining both structural barriers and the extent to which ethical considerations are operationalized.^134^ These barriers are consistent with broader findings in clinical AI adoption, where workflow integration, trust, and organizational readiness are identified as key determinants of implementation success.^135^

**Figure 8:**
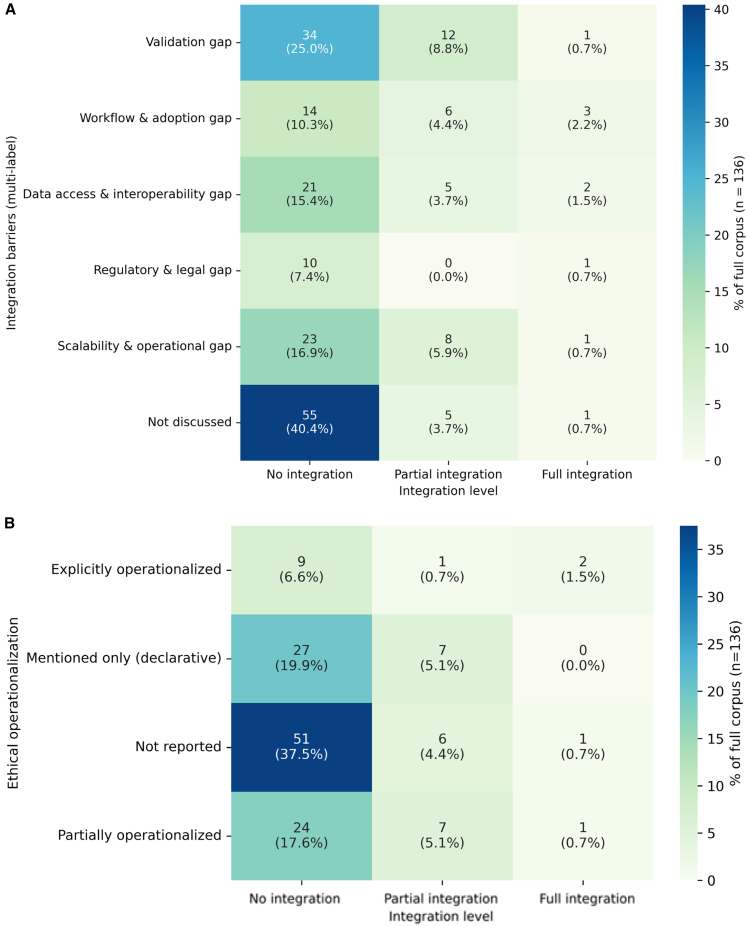
Integration barriers and ethical operationalization across clinical integration levels in HRSs (*n* = 136) (A) Heatmap showing the distribution of reported integration barriers (multi-label) across clinical integration levels. Validation, scalability, and interoperability gaps are predominantly associated with systems lacking clinical integration, while barriers are more explicitly reported in partially integrated systems. (B) Heatmap showing the distribution of ethical operationalization levels across clinical integration levels. A clear gradient emerges, with limited ethical mechanisms in non-integrated systems and more explicit operationalization in more integrated ones. (A) and (B) indicate that technical and organizational barriers, along with ethical operationalization, evolve with clinical integration but remain unevenly addressed. This misalignment reinforces the structural nature of the translation gap.

The relationship between reported barriers and clinical integration levels (Figure 8A) indicates that systems without integration are predominantly associated with unreported or unresolved challenges, with a substantial portion of studies not explicitly documenting integration barriers.^136^ Among reported barriers, validation gaps are the most prevalent,^137^ followed by scalability constraints^138^ and data access or interoperability limitations.^139^ These results suggest that the absence of clinical integration reflects the accumulation of unresolved technical, organizational, and evaluative challenges, rather than a single limiting factor.

As integration increases, barrier profiles evolve. Systems with partial integration more frequently report validation, workflow, and scalability constraints, indicating that engagement with clinical contexts exposes the challenges not apparent at the prototype stage. Fully integrated systems remain rare, and their reported barriers are limited, reflecting both the difficulty of achieving this level and the scarcity of detailed reporting.

Ethical operationalization follows a parallel but uneven trajectory (Figure 8B). Systems without clinical integration predominantly lack explicit ethical mechanisms or rely on high-level declarative statements. In contrast, partially and fully integrated systems more frequently report concrete ethical considerations, although these remain inconsistently implemented. Even among deployed systems, the operationalization of ethical principles—such as transparency, accountability, and fairness—remains limited.^101^ This gap aligns with recent evidence showing that explainable AI, when operationalized within clinical systems, can act as a practical enabler of trust and adoption, rather than a purely theoretical requirement.^140^

Taken together, these findings indicate that clinical integration and ethical operationalization evolve in parallel but not in synchrony. Overcoming technical and organizational barriers is necessary for integration but does not guarantee ethically robust deployment. This misalignment highlights that translational maturity in HRSs should be assessed not only through deployment and workflow integration but also through the explicit operationalization of ethical, legal, and governance principles.

#### Cross-pillar patterns shaping translational maturity

While previous sections examine each lifecycle pillar of an HRS independently, real-world translation cannot be explained by any single dimension in isolation. Translational maturity instead emerges from the alignment—or misalignment—between intended use, evaluation strategy, clinical integration, and ethical operationalization. To capture this systemic perspective, we adopt a cross-pillar synthesis that links design intent, validation depth, and governance readiness into a unified analytical lens.

By jointly analyzing attributes across pillars, this approach distinguishes systems whose limited deployment reflects design-aligned research positioning from those where stagnation signals genuine translational bottlenecks. Rather than treating deployment outcomes as uniformly indicative of failure, maturity is interpreted relative to intended use and the degree to which technical validation is complemented by contextual and organizational grounding.

##### Intended use as a structuring driver of translational maturity

**Figure 9:**
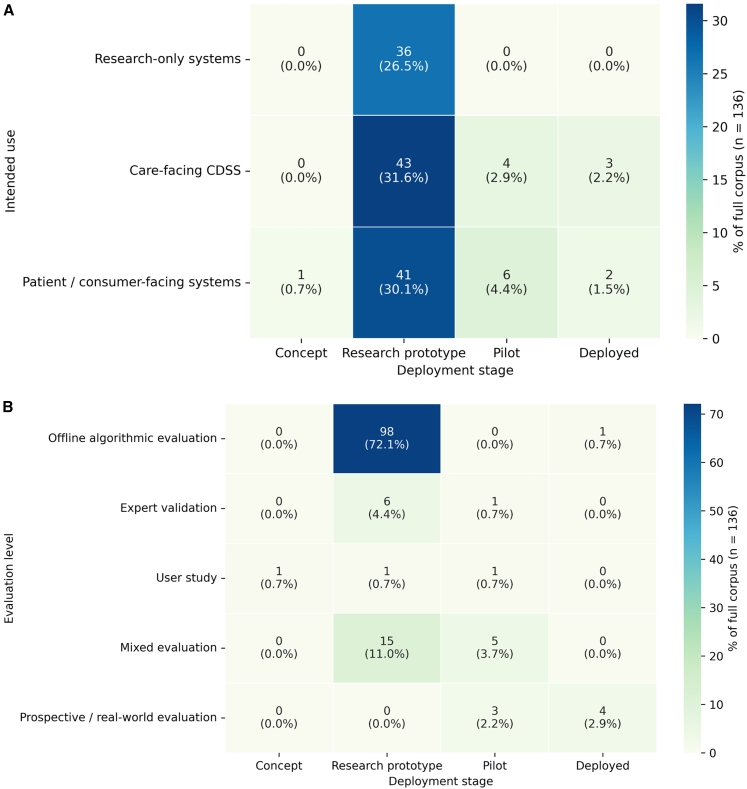
Cross-pillar patterns linking intended use, evaluation strategy, and deployment stage in HRSs (*n* = 136) (A) Heatmap showing the distribution of intended use categories across deployment stages. Research-oriented systems remain confined to the research prototype stage, whereas care- and patient-facing systems show limited progression toward pilot and deployed stages. (B) Heatmap showing the distribution of evaluation levels across deployment stages. Offline algorithmic evaluation is concentrated at the research prototype stage, while pilot and deployed systems are more often associated with mixed or real-world evaluation. (A) and (B) indicate that translational progression in HRSs is jointly conditioned by intended use and evaluation depth, with offline-only validation limiting advancement beyond the prototype stage.

Deployment stage is often used as a proxy for translational success, yet its interpretation depends critically on intended use. The joint distribution of intended use and deployment stage (Figure 9A) reveals three distinct trajectories.

First, research-oriented systems remain entirely confined to the research prototype stage. This reflects a coherent alignment between intent and realization, where deployment is not a design objective but rather supports methodological exploration and hypothesis generation. In this context, limited deployment represents conceptual completeness, rather than translational deficiency.

Second, care-facing CDSSs exhibit a clear misalignment. Despite being explicitly designed to support clinical decision-making, the majority remains at the prototype stage,^30^ with only limited progression to pilot^26^ and rare to deployment.^115^ This gap reflects not only the technical limitations but also deeper constraints related to validation requirements, workflow integration, regulatory oversight, and accountability structures.

Third, patient- or consumer-facing systems occupy an intermediate position. While most remain prototypes, pilot and deployed instances are more frequent than in care-facing systems, likely reflecting lower regulatory thresholds and more flexible deployment contexts. However, their limited progression indicates that reduced regulatory burden alone is insufficient to ensure translation, pointing instead to persistent challenges in sustained evaluation and user-centered validation.

Together, these trajectories demonstrate that translational maturity is inherently contextual and cannot be inferred from deployment stage alone but must be interpreted relative to system intent.

##### Evaluation strategy as a structural bottleneck to deployment

Evaluation strategy emerges as a primary cross-pillar determinant of translational progression. The joint analysis of evaluation level and deployment stage (Figure 9B) reveals a highly constrained pathway linking validation practices to deployment outcomes.

The dominant configuration corresponds to offline algorithmic evaluation at the research prototype stage, accounting for over 70% of studies.^53^^,^^141^^,^^142^ While appropriate for early-stage development, this approach creates a structural ceiling: systems validated exclusively through offline metrics rarely progress toward pilot testing or real-world deployment.

In contrast, human-centered evaluation strategies—including expert validation, user studies, and mixed approaches—are disproportionately associated with systems that advance beyond prototypes.^103^^,^^122^ Mixed evaluation, in particular, appears as a transitional layer that bridges algorithmic validation and contextual feasibility, enabling gradual exposure to real-world constraints.

Notably, prospective or real-world evaluation appears only at the pilot or deployed stage, indicating that such validation is introduced late rather than being progressively integrated. This discontinuity suggests that evaluation depth and deployment maturity evolve in a tightly coupled yet non-linear manner, where progression requires a qualitative shift in validation paradigm, rather than incremental refinement.

These findings position evaluation strategy as a central bottleneck in the HRS lifecycle. For care- and patient-facing systems, the persistence of offline-only validation reflects a critical misalignment between intended use and evidentiary support, directly constraining real-world adoption.

##### Cross-pillar drivers of translational maturity

Across the corpus, translational maturity is shaped by recurrent structural dependencies spanning multiple dimensions. Systems positioned as care-facing or patient-facing ones frequently rely on offline evaluation and remain confined to the prototype stage,^97^^,^^143^^,^^144^ indicating a systematic misalignment between intended use and validation depth.

A second pattern links clinical integration with organizational readiness. Systems reporting no or partial integration often lack explicit workflow alignment, scalability planning, or integration pathways (Figure 8A), whereas systems approaching pilot testing or deployment more frequently report intermediate validation mechanisms, stakeholder involvement, and early integration efforts. This suggests that integration readiness co-evolves with validation strategy, rather than emerging as a downstream outcome.

Finally, ethical and governance attributes exhibit a similar trajectory. Explicit ethical operationalization and governance mechanisms are rarely reported in early-stage systems but become more visible in advanced stages, suggesting that trust, accountability, and transparency are progressively articulated as systems approach real-world use.

Overall, translational maturity in HRSs does not arise from isolated technical performance but from the coordinated alignment of intended use, evaluation strategy, clinical integration, and ethical operationalization. These cross-pillar dependencies reveal that translation is not a linear progression but a systemic configuration problem, providing a structured foundation for the conceptual synthesis developed in discussion.

## Discussion

The findings of this review reveal a consistent and structurally grounded pattern across the HRS literature: despite increasing algorithmic sophistication and the expanding application scope, most systems remain positioned at early stages of translational maturity. Rather than interpreting this as a limitation of recommender technologies in healthcare, the results call for reframing how translation is conceptualized and assessed.

This section synthesizes cross-dimensional patterns identified across the system lifecycle and argues that limited real-world adoption is best understood as a problem of systemic alignment—between design intent, evaluation strategy, clinical integration pathways, and governance readiness—rather than as a consequence of insufficient technical capability.

### Translational maturity as alignment, not progression

A dominant assumption in digital health equates translational success with deployment status, implicitly treating non-deployed systems as immature. The findings challenge this linear view. By jointly examining intended use, evaluation strategy, and deployment stage, it becomes clear that limited deployment does not have a uniform meaning across HRSs.

For research-oriented systems, confinement to the prototype stage reflects a coherent alignment between intent and realization (Figure 9A). These systems are explicitly framed as methodological explorations or algorithmic testbeds, without claims of operational use. In this context, non-deployment is not failure, but consistency.

In contrast, care-facing and patient-facing systems reveal a more constrained pattern. Although they explicitly target decision support, they largely remain at early deployment stages. As shown in Figures 9A and 9B, this positioning frequently co-occurs with the evaluation strategies limited to offline or retrospective validation. This reveals a misalignment between intended use and validation depth, suggesting that translational limitations stem from lifecycle incoherence rather than technical insufficiency.

Taken together, these observations indicate that translational maturity cannot be inferred from deployment alone. It emerges from the coherence between system purpose, evaluation design, and the conditions under which recommendations can be meaningfully enacted. Translation, therefore, is not a linear progression, but an alignment-dependent state.

### Evaluation strategy as a structural bottleneck

Evaluation strategy emerges as a central determinant of translational positioning. Offline algorithmic validation dominates the literature and is strongly associated with research prototype systems. While appropriate for early-stage development, it constrains downstream applicability by isolating performance from clinical workflows, user interpretation, and institutional constraints.

Systems progressing beyond the prototype stage are disproportionately associated with human-centered validation, including expert review, user studies, and mixed designs. These approaches are primarily observed at the pilot level, suggesting that stakeholder engagement is not a refinement but a prerequisite for translation. Evaluation strategy, thus, functions as a structural gatekeeper: systems validated solely as algorithmic artifacts remain confined to methodological spaces.

Moreover, a prospective or real-world evaluation appears only in pilot and deployed systems (Figure 9B), indicating a discontinuity rather than a gradual transition. Evaluation depth and deployment maturity, therefore, evolve in a tightly coupled, non-linear manner.

These findings reframe translational stagnation in HRSs: systems do not fail to deploy because they underperform technically, but because their evaluation strategies do not position them to cross clinical and organizational thresholds.

### Integration, governance, and accountability as enabling conditions

Beyond evaluation, translational positioning is consistently associated with the explicit articulation of integration pathways, governance mechanisms, and accountability structures. Systems approaching pilot or deployed stages more frequently report workflow alignment, human oversight, explainability, and ethical safeguards, whereas research-stage systems tend to omit these dimensions or address them superficially.

These patterns can be interpreted through the lens of established implementation science frameworks, such as the Consolidated Framework for Implementation Research (CFIR) and the Non-adoption, Abandonment, Scale-up, Spread, Sustainability (NASSS), which emphasize the role of workflow integration, organizational readiness, and trust as critical determinants of successful clinical adoption. This perspective is further supported by recent empirical evidence on AI deployment in healthcare, highlighting that explainability and alignment with clinical practices are central to overcoming translational barriers.^135^^,^^140^

While this pattern reflects differences in reporting rather than confirmed absence, explicit articulation remains a critical proxy for readiness to operate in regulated environments. From a translational perspective, what is not reported is unlikely to be operationalized.

In this sense, governance, explainability, and HITL configurations are not late-stage constraints, but structural enablers. Their co-occurrence with higher integration levels suggests that ethical and organizational readiness is intrinsically linked to trust, institutional acceptance, and responsibility allocation.

### Implications for future HRS research

Taken together, these findings suggest that the central challenge in HRS research is no longer technical capability, but structural positioning for accountable use. Translational readiness should be understood as a composite property emerging from alignment across lifecycle dimensions, rather than as a binary outcome defined by deployment status.

Three implications follow:

First, evaluation strategies must be aligned with intended use from the earliest stages of development. Systems designed for direct patient support or clinician-facing decisions require validation strategies capable of supporting that level of responsibility. Offline validation alone is insufficient for systems claiming real-world decision support.

Second, reporting standards must move beyond predictive performance and include explicit articulation of decision authority, workflow assumptions, governance mechanisms, and implementation boundaries. Without these dimensions, translational claims remain conceptually weak regardless of technical sophistication.

Third, system maturity should be assessed through cross-dimensional coherence, rather than isolated performance indicators. A technically advanced model with weak evaluation and no integration pathway is less translationally mature than a simpler system with strong contextual alignment and validated operational use.

This perspective shifts the discourse from technological innovation to lifecycle design. Bridging the gap between methodological excellence and real-world impact will require not incremental performance gains, but a deliberate reorientation toward use-centered, governance-aware, and clinically integrated system development.

The contribution of this review, therefore, extends beyond descriptive synthesis. By reframing translational maturity as a problem of structural coherence rather than algorithmic limitation, it provides a diagnostic lens for interpreting the current HRS landscape and a strategic foundation for future research. HRSs are more likely to reach clinical practice not by becoming more complex, but by becoming better aligned with the environments in which they are expected to function.

### Limitations of the study

While this review provides a comprehensive and lifecycle-oriented synthesis of HRSs, several methodological considerations should be acknowledged when interpreting the findings.

First, the analysis relies exclusively on information explicitly reported in published studies. Dimensions such as clinical integration, governance, explainability, and HITL configurations were coded based on authors’ descriptions, rather than inferred system properties. As such, the absence of reporting should not be interpreted as the absence of implementation. From a translational perspective, reporting itself constitutes a proxy for transparency and accountability, meaning that observed gaps reflect limitations in both reporting practices and system articulation.

Second, the search strategy was centered on the “recommender system” terminology to ensure conceptual coherence. However, this focus may have reduced sensitivity to adjacent domains, particularly CDSSs and AI-based decision tools that implement recommendation functionalities under different labels. This reflects a trade-off between conceptual specificity and coverage breadth and should be considered when interpreting the scope of the findings.

Third, the classification framework simplifies a heterogeneous and evolving field. Categories such as clinical integration, intended use, and ethical operationalization capture dominant orientations, rather than exhaustive implementation realities. Coding was based on explicit *a priori* definitions and conservative attribution rules, with ambiguous cases classified as “not reported” to avoid overinterpretation.

Fourth, the exclusion of conference proceedings and the focus on journal articles may bias the corpus toward more mature research outputs, particularly in a field where early innovations are often reported in computer science venues. Future work could extend this framework to high-quality conference literature.

Fifth, the proposed lifecycle framework should be understood as an analytical lens rather than a prescriptive pipeline. It does not imply causal or temporal dependencies but provides a structured way to examine coherence and misalignment across system design, evaluation, and translational positioning.

Finally, this review emphasizes translational alignment rather than algorithmic performance and does not quantitatively compare models or techniques. Complementary meta-analyses could extend this perspective by focusing on performance evaluation.

Taken together, these considerations clarify the analytical scope and reinforce the relevance of a lifecycle perspective for understanding why many HRSs remain confined to experimental stages despite substantial technical progress.

## Resource availability

### Lead contact

Requests for further information and resources should be directed to and will be fulfilled by the lead contact, Oumaima EL MIAYAR (o.elmiayar@research.emi.ac.ma).

### Materials availability

This study did not generate new unique materials.

### Data and code availability

• The dataset generated during this study has been deposited in Mendeley Data and is publicly accessible at https://doi.org/10.17632/krfvhrkwpn.1.
• This paper does not report original code.
• Any additional information required to reanalyze the data reported in this paper is available from the lead contact upon request.

## Acknowledgments

This work was supported by the 10.13039/501100002385Ministry of Higher Education, Scientific Research and Innovation; the Digital Development Agency; and the National Center for Scientific and Technical Research (CNRST) of Morocco, under the Al Khawarizmi program.

## Author contributions

Conceptualization, O.E.M and A.B.; methodology, investigation, data curation, formal analysis, visualization, and writing – original draft, O.E.M.; writing – review & editing, O.E.M. and A.B.; supervision and project administration, A.B.

## Declaration of interests

The authors declare no competing interests.

## Declaration of generative AI and AI-assisted technologies in the writing process

During the preparation of this work, the authors used OpenAI’s ChatGPT to support language editing and manuscript refinement. After using this tool, the authors carefully reviewed and edited the content as needed, and they take full responsibility for the content of the published article.

## STAR★Methods

### Key resources table

**Table:** 

REAGENT or RESOURCE	SOURCE	IDENTIFIER
**Deposited data**		

Extracted dataset of included studies	Mendeley Data	https://doi.org/10.17632/krfvhrkwpn.1

**Other**		

Scopus database	Elsevier	https://www.scopus.com
PubMed database	NCBI	https://pubmed.ncbi.nlm.nih.gov
PRISMA 2020 guidelines	Page et al.^10^	https://doi.org/10.1136/bmj.n71

### Method details

#### Review design and protocol

This study adopts a systematic literature review (SLR) methodology aligned with the PRISMA 2020 guidelines,^10^ ensuring transparency, reproducibility, and methodological rigor. The review is grounded in a lifecycle-oriented analytical framework designed to examine how Health Recommender Systems (HRS) are conceptualized, evaluated, and positioned for real-world clinical translation. No prior protocol was registered.

#### Research questions and scope

This SLR provides a lifecycle-oriented synthesis of Health Recommender Systems, focusing on the interplay between system design, evaluation practices, and translational positioning across healthcare contexts. Rather than examining isolated technical components, the review adopts an integrative perspective to capture how multiple dimensions jointly shape system maturity and real-world readiness.

The study is guided by the following overarching research question: How are Health Recommender Systems designed, evaluated, and positioned for clinical translation, and what structural patterns explain the gap between technical development and real-world integration?

To operationalize this objective, six analytical dimensions are considered: (1) clinical domains, health tasks, target users, and intended use; (2) data sources, sensitivity, governance, and reproducibility practices; (3) recommendation paradigms, output types, and explainability mechanisms; (4) system delivery modes, interaction patterns, and human oversight; (5) evaluation strategies and outcome maturity; and (6) clinical integration, deployment progression, reported barriers, and ethical operationalization.

#### Search strategy

A systematic literature search was conducted using two complementary databases, Scopus and PubMed, to ensure broad interdisciplinary coverage spanning healthcare, biomedical informatics, and computational systems. The databases were last searched in December 2025.

Two complementary free-text queries were used: (“recommender system∗” OR “recommendation system∗“) AND “healthcare”, and (“health recommender∗” OR “digital health recommender∗” OR “personalized health recommendation∗” OR “personalised health recommendation∗“). Retrieved records were merged and deduplicated prior to screening.

The review includes peer-reviewed journal articles published between 2014 and 2025 in English. Conference papers were excluded to prioritize methodological completeness and evaluation rigor, although this may limit early-stage contributions.

#### Eligibility criteria

Studies were included if they implemented recommender systems in healthcare and described a recommendation process involving personalized suggestions, decision support, or adaptive guidance.

Studies were excluded if they focused exclusively on prediction or classification without an explicit recommendation component, addressed non-health domains, or lacked sufficient methodological transparency to support reliable data extraction.

#### Study selection process

Study selection followed a two-stage screening process (title/abstract followed by full-text review). Screening was conducted by a single reviewer, which represents a methodological limitation. To mitigate this, an independent validation was performed on a subset of studies using inter-rater reliability assessment. The overall process is summarized in the PRISMA flow diagram (Figure 1).

#### Data extraction and analytical framework

Data extraction was conducted using a structured template based on six analytical pillars: (1) Clinical Need and Use Context; (2) Data Acquisition and Governance; (3) Recommendation Logic and Output; (4) User Experience and Interaction; (5) Evaluation and Validation Strategy; and (6) Translation, Integration, and Ethics.

The life cycle perspective is used as an analytical lens to examine HRS across interconnected stages of design, evaluation, interaction, and real-world translation.

Each attribute was defined using explicit operational rules and associated with predefined value sets to ensure coding consistency while accommodating heterogeneity. Multi-label coding was applied when multiple values were explicitly reported, while single-label coding was used when a dominant category could be identified. When information was not reported, the category “Not reported” was assigned to preserve transparency. A detailed description of attributes is provided in Table S1.

#### Coding procedure and reliability

Data extraction and coding were conducted at the full-text level using a standardized template, based strictly on reported information.

To assess coding consistency, a subset of 30 studies (approximately 22%) was independently evaluated by a second reviewer for key translational attributes (deployment stage and clinical integration level) (Tables S2a and S2b). Inter-rater reliability was assessed using Cohen’s kappa coefficient. The results indicate almost perfect agreement for deployment stage (κ = 0.87) and substantial agreement for clinical integration (κ = 0.77), supporting the robustness and internal consistency of the coding framework (Table S2c). The remaining studies were coded using predefined criteria, with conservative rules applied to avoid overinterpretation.

#### Quality, bias, and certainty considerations

No formal risk-of-bias assessment tool was applied. This reflects the descriptive and structural objective of the review, which aims to characterize HRS design and translational positioning rather than evaluate intervention effectiveness. Study quality was instead considered indirectly through reporting completeness and methodological transparency.

The analysis relies on explicitly reported information and is therefore sensitive to reporting practices. No formal reporting bias assessment was conducted.

The certainty of evidence was not formally evaluated, as the review focuses on structural mapping rather than outcome synthesis.

### Quantification and statistical analysis

Data synthesis followed a mixed quantitative and qualitative approach, focusing on the structural characterization and translational positioning of Health Recommender Systems rather than outcome comparison. Accordingly, no effect measures were defined.

Studies were grouped based on shared attributes across the life cycle framework, enabling structured comparison of system design, evaluation strategies, interaction modalities, and clinical integration levels.

Descriptive statistics (frequencies and proportions with 95% Wilson confidence intervals) were computed to summarize the distribution of extracted attributes. When information was not explicitly reported, the category “Not reported” was assigned.

Results were presented through structured tables and cross-dimensional figures in the main text and supplementary materials. Due to heterogeneity in study designs and objectives, no meta-analysis was performed. Instead, synthesis relied on comparative and cross-dimensional analysis to identify patterns and gaps across the HRS life cycle.

Variability across studies was explored through cross-dimensional comparisons across the six analytical pillars, rather than statistical heterogeneity analysis.

No formal sensitivity analysis was conducted; however, robustness was supported through conservative coding rules and inter-rater reliability assessment.

Finally, a qualitative interpretive synthesis was conducted to contextualize the quantitative findings and provide a structured understanding of HRS design, evaluation, and translational readiness.
