# Supplementary material for: Why health recommender systems struggle to reach clinical practice: A lifecycle-oriented systematic review
Source: iScience. 2026 Jun 9;29(6):116220. doi: 10.1016/j.isci.2026.116220 (PMC13273477; doi:10.1016/j.isci.2026.116220)
Supplement: Document S1. Tables S1, S2a, and S2c [file mmc1.pdf]

iScience, Volume *29*

Supplemental information

Why health recommender systems struggle to reach clinical practice: A lifecycle-oriented systematic review

Oumaima EL MIAYAR and Abdelaziz Berrado

Supplemental Information

S1. Analytical framework

Table S1. Analytical framework: pillars, attribute definitions, and coding values used in the study, related to STAR Methods

Pillar	Attribute (ID)	Definition	Coding Values
**P1. Clinical Need and Use Context**	Clinical Domain (A1)	Medical or health domain addressed by the HRS	General health & wellness; Chronic diseases; Cancer; Mental & neurological health; Infectious diseases; Acute/hospital care; Oral & dental health; Multi-disease
	Clinical Task (A2)	Primary healthcare task supported by the system	Diagnosis/risk prediction; Treatment recommendation; Prevention & lifestyle support; Monitoring & follow-up; Information/navigation; Research & discovery
	Target User (A3)	Intended end-user of the system	Clinician; Patient; Dual (clinician + patient); Consumer/non-clinical
	Intended Use (A4)	Functional positioning of the system	Research-only systems; Care-facing CDSS; Patient-/consumer-facing health support
**P2. Data Acquisition and Governance**	Data Source Type (A5)	Origin of data used for recommendation	Public/open datasets; Retrospective clinical data; Patient-generated data; Sensor/IoT data; Synthetic/simulated data; Mixed sources; Not reported
	Data Sensitivity (A6)	Sensitivity level of the data as reported	Sensitive; Non-sensitive; Not reported
	Governance Reporting (A7)	Reporting of ethical, legal, or governance mechanisms	Ethics/consent statement; Technical governance mechanisms; Regulatory compliance (GDPR/HIPAA); Not reported
	Reproducibility Signal (A8)	Availability of data and/or code	Data available; Code available; Data & code available; Not applicable; Not reported
**P3. Recommendation Logic and Output**	Recommendation Paradigm (A9)	Core algorithmic logic of the recommender	Knowledge-based; Content-based; Collaborative filtering; ML/DL-based; Graph-based; Simulation-based; Hybrid; Not reported
	Output Type (A10)	Form of recommendation output	Ranked list; Risk score/prediction; Decision policy/care plan; Narrative advice; Alerts/flags
	Explainability Level (A11)	Degree of explainability provided	Post-hoc explainability; Intrinsic explainability; Evidence-linked/knowledge-grounded; Not reported
**P4. User Experience, Interaction, and Delivery**	Delivery Platform (A12)	Medium through which recommendations are delivered	Web-based; Mobile-based; Web–mobile; EHR/CDSS-integrated; Conversational/chatbot; Not reported
	Interaction Mode (A13)	Degree of user interaction with the system	Interactive; Passive; Not reported
	Human-in-the-Loop Level (A14)	Degree of human decision authority	No human involvement; Human review; Final human decision authority; Not reported
**P5. Evaluation and Validation Strategy**	Evaluation Level (A15)	Type of evaluation conducted	Offline algorithmic evaluation; Expert validation; User study; Mixed evaluation; Prospective/real-world

Continued on next page

Pillar	Attribute (ID)	Definition	Coding Values
	Outcome Type (A16)	Nature of evaluated outcomes	Technical performance; Clinical outcomes; User-centered outcomes; Mixed outcomes
**P6. Translation, Integration, and Ethics in Practice**	Clinical Integration Level (A17)	Degree of integration into clinical workflows	None; Partial; Full
	Deployment Stage (A18)	Maturity of system deployment	Concept; Research prototype; Pilot; Deployed
	Integration Barriers (A19)	Reported barriers to real-world integration	Validation; Workflow compatibility; Scalability; Data access/interoperability; Regulatory; Not reported (Not discussed)
	Ethical Operationalization (A20)	Degree of ethical implementation beyond declarative statements	Declarative only; Partial operationalization; Explicit operationalization; Not reported

S2. Inter-rater Reliability Analysis

To ensure the robustness, consistency, and reproducibility of the annotation process, inter-rater reliability was assessed on a subset of 30 studies independently coded by two reviewers. Agreement was evaluated for two key variables: *Deployment Stage* and *Clinical Integration*. Cohen's kappa coefficient was used to quantify agreement beyond chance.

Table S2a. Inter-rater annotations for Deployment Stage (n = 30), related to STAR Methods

Paper	Reviewer 1	Reviewer 2
P1	Research prototype	Research prototype
P2	Pilot	Pilot
P3	Deployed	Deployed
P4	Deployed	Deployed
P5	Deployed	Deployed
P6	Deployed	Deployed
P7	Deployed	Pilot
P8	Pilot	Pilot
P9	Pilot	Pilot
P10	Research prototype	Research prototype
P11	Research prototype	Pilot
P12	Research prototype	Research prototype
P13	Research prototype	Research prototype
P14	Research prototype	Research prototype
P15	Research prototype	Research prototype
P16	Research prototype	Research prototype
P17	Pilot	Pilot
P18	Concept	Concept
P19	Pilot	Pilot
P20	Research prototype	Research prototype
P21	Research prototype	Research prototype
P22	Research prototype	Research prototype
P23	Research prototype	Research prototype
P24	Research prototype	Research prototype
P25	Pilot	Pilot
P26	Research prototype	Research prototype
P27	Research prototype	Research prototype
P28	Research prototype	Research prototype
P29	Research prototype	Research prototype
P30	Research prototype	Research prototype

Table S2b. Inter-rater annotations for Clinical Integration (n = 30), related to STAR Methods

Paper	Reviewer 1	Reviewer 2
P1	No clinical integration	No clinical integration
P2	Partial clinical integration	Partial clinical integration
P3	Full clinical integration	Full clinical integration
P4	Full clinical integration	Full clinical integration
P5	Partial clinical integration	Partial clinical integration
P6	No clinical integration	No clinical integration
P7	Full clinical integration	Full clinical integration
P8	No clinical integration	No clinical integration
P9	Partial clinical integration	Partial clinical integration
P10	No clinical integration	No clinical integration
P11	Partial clinical integration	Partial clinical integration
P12	Partial clinical integration	Partial clinical integration
P13	Partial clinical integration	No clinical integration
P14	No clinical integration	No clinical integration
P15	No clinical integration	No clinical integration
P16	No clinical integration	No clinical integration
P17	No clinical integration	No clinical integration
P18	No clinical integration	No clinical integration
P19	No clinical integration	No clinical integration
P20	Partial clinical integration	Partial clinical integration
P21	No clinical integration	No clinical integration
P22	No clinical integration	No clinical integration
P23	No clinical integration	No clinical integration
P24	No clinical integration	No clinical integration
P25	No clinical integration	Partial clinical integration
P26	Partial clinical integration	No clinical integration
P27	No clinical integration	No clinical integration
P28	No clinical integration	No clinical integration
P29	No clinical integration	No clinical integration
P30	No clinical integration	No clinical integration

Table S2c. Summary of Cohen's kappa results, related to STAR Methods

Variable	P_o	P_e	κ
Deployment Stage	0.933	0.485	0.87
Clinical Integration	0.90	0.56	0.77

Interpretation:

The Cohen's kappa value for *Deployment Stage* ($\kappa = 0.87$) indicates almost perfect agreement, demonstrating a high level of consistency and robustness in the annotation process.

The Cohen's kappa value for *Clinical Integration* ($\kappa = 0.77$) indicates substantial agreement.
